# Benchmarking Psychiatry in Europe and beyond: The European Board Exam of Psychiatry

**DOI:** 10.1192/j.eurpsy.2024.1746

**Published:** 2024-04-24

**Authors:** Andrew Brittlebank, Livia De Picker, Krzysztof Krysta, Asilay Seker, Ilaria Riboldi, Cecile Hanon, George Alexandru Stercu, Egor Chumakov, Jerzy Samochowiec, Geert Dom, Marisa Casanova Dias

**Affiliations:** 1 Union Européenne des Médecins Spécialistes (UEMS) Section of Psychiatry, Brussels, Belgium; 2 SINAPS, University Psychiatric Hospital Campus Duffel, Duffel, Belgium; 3 Collaborative Antwerp Psychiatric Research Institute, Faculty of Medicine, University of Antwerp, Antwerp, Belgium; 4 Department and Clinic of Rehabilitation Psychiatry, Faculty of Medical Sciences in Katowice, Medical University of Silesia in Katowice, Katowice, Poland; 5 Institute of Psychiatry, Psychology & Neuroscience, King’s College London, London, UK; 6 South London and Maudsley NHS Foundation Trust, London, UK; 7 Department of Medicine and Surgery, University of Milano-Bicocca, Monza, Italy; 8 Regional Resource Center of Old Age Psychiatry, AP-HP, Centre – University of Paris, Psychiatry and Addictology Department, Corentin-Celton Hospital, Issy-les-Moulineaux, France; 9 Prof. Dr. Alexandru Obregia Hospital, Bucharest, Romania; 10 Department of Psychiatry and Addiction, Saint-Petersburg State University, St Petersburg, Russia; 11 Department of Psychiatry, Pomeranian Medical University, Szczecin, Poland; 12 PC Multiversum, Boechout, Belgium; 13 European Psychiatric Association, Brussels, Belgium; 14 National Centre for Mental Health, Cardiff University, Cardiff, UK; 15 Section of Women’s Mental Health, Institute of Psychiatry, Psychology & Neuroscience, King’s College London, London, UK

In 2021, the European Psychiatric Association (EPA), the Section of Psychiatry of the European Union of Medical Specialists (UEMS), and the European Federation of Psychiatric Trainees (EFPT) took the decisive step to formally join forces in collaborating toward the development and implementation of a European Board Exam of Psychiatry. The EPA (https://www.europsy.net/) is the main scientific organization representing national psychiatric associations in Europe. The UEMS (https://www.uemspsychiatry.org/) is the professional organization mandated with setting standards and educational accreditation authority within Europe. The EFPT (https://efpt.eu/) is the organization representing the consensus of psychiatric trainees’ associations across European countries.

Two reasons compel us to establish a European Board Exam of Psychiatry: first, to influence the learning outcomes of future psychiatric specialists, and second, to harmonize professional standards across Europe. Both outcomes are necessary to ensure high-quality mental healthcare. As Schuwirth and van der Vleuten observed, “students do what you inspect,” in other words, what is learned is determined by what and how the subject is assessed [1]. Because psychiatry is a deeply clinical specialty, it must be recognized that a written knowledge examination cannot possibly assess all that is required of a psychiatrist. Nonetheless, it sets a foundational benchmark for aspiring specialists. Furthermore, the harmonization of medical education and training is critical for ensuring high-quality healthcare and facilitating the mobility of healthcare professionals within Europe. This does not imply that training programs should be equal, ignoring local and cultural distinctions. Instead, harmonization seeks to establish a strong base of fundamental training prerequisites, allowing for the addition of country-specific aspects, and leveling up training standards. European Board Exams are central to this objective, establishing benchmarks for the required knowledge, skills, and competencies of medical professionals that complement the national evaluation procedures. In Europe, there are UEMS exams in 34 medical specialty fields. Existing European Board Exams in ophthalmology [2], anesthesiology [3], radiology [4], and gastroenterology [5] managed by the UEMS have demonstrated the value of a structured examination framework that integrates theoretical and practical skill assessments. The Board Exam of Psychiatry will benefit from these precedents, adopting best practices in examination content, fairness, and delivery technologies, while also addressing European diversity and maintaining examination integrity.

**Figure 1.**
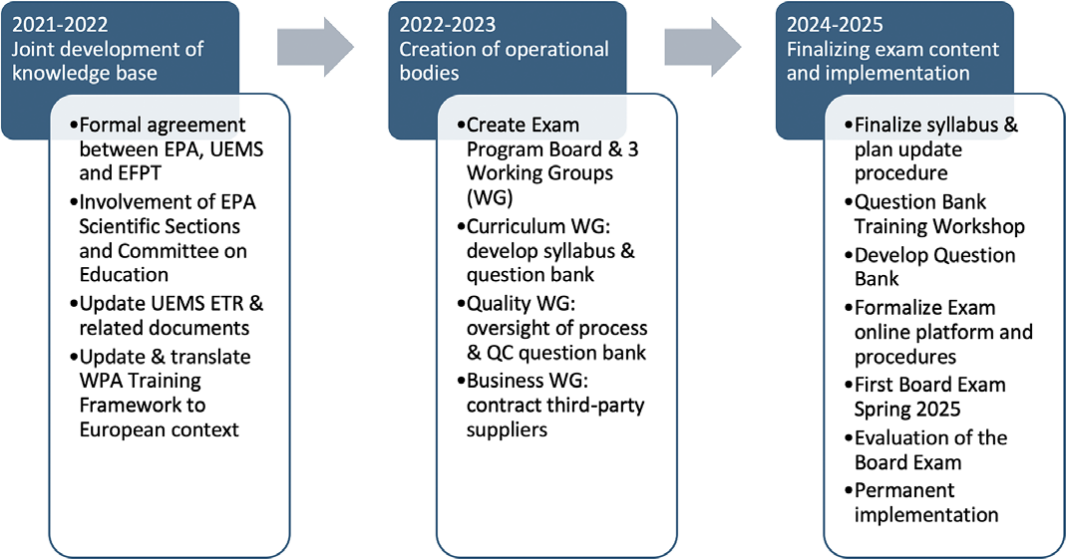
Workflow and timeline of the European Psychiatry Board Exam.

Accordingly, an Exam Program Board, representing the leadership of the three acting associations, was created and tasked with overseeing the project’s coordination. A structured timeline was established, targeting the first exam at the beginning of 2025 (Figure 1).

This process built upon the experiences of other medical specialty fields, where cooperation between European scientific associations and the UEMS, has proven successful. A unique feature of the Board Exam of Psychiatry is the inclusion of diverse stakeholder perspectives from its inception, particularly from groups directly impacted by the exam, such as psychiatric trainees and early career psychiatrists through the representation of these groups in the Exam Program Board by the EFPT and the EPA Early Career Psychiatrists’ Committee (ECPC). To the best of our knowledge, this is the first example of a European Board Exam where the potential exam takers actively participate in its organization, ensuring careful consideration of their needs.

**Figure 2.**
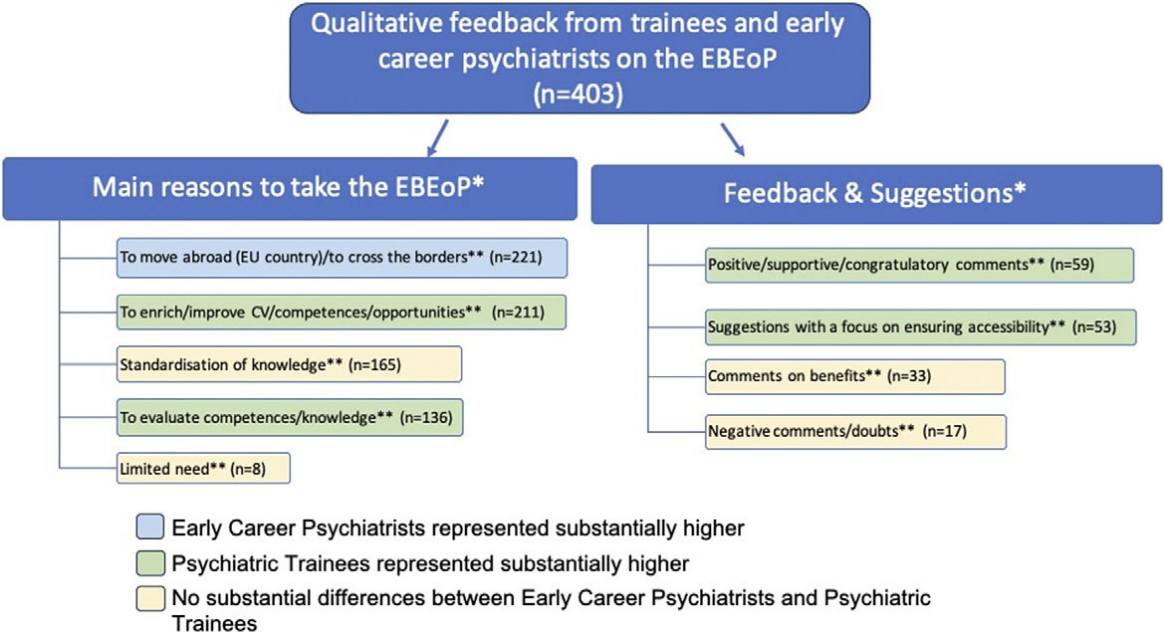
Thematic analysis of the qualitative feedback of trainees and early career psychiatrists on a Board Exam of Psychiatry. EBEoP, European Board Exam of Psychiatry; *, Themes; **, Subthemes.

As part of the initial stakeholder input, the EFPT and the EPA ECPC collaboratively collected qualitative feedback from over 400 psychiatric trainees and early career psychiatrists from 40 European countries on their views, attitudes, and expectations regarding the Board Exam of Psychiatry. This feedback, analyzed through Thematic Analysis, overwhelmingly supported the creation of the examination and informed stages of its development. Specific themes and subthemes that emerged are summarized in Figure 2.

A knowledge exam requires a comprehensive content base (i.e., a syllabus). The Board Exam of Psychiatry’s syllabus is based on the revised UEMS European Training Requirements (ETR) approved in 2023 [6], and encompasses basic and advanced training needs and competencies framed within the CanMEDS model [7]. Additional content expertise is provided by the EPA Sections and external specialists, with educational oversight by the UEMS Section on Psychiatry and the EPA Committee on Education. Financially, the EPA and UEMS have committed to supporting the initial implementation for two consecutive exams (2025 and 2026), with the goal of achieving financial sustainability through future examination revenues.

Scheduled to commence in 2025, the examination will be a knowledge-based test, in written format, conducted online, in English, and under strict proctoring to ensure its integrity and fairness. This is a voluntary, supplemental examination that can be taken by candidates who hold a Primary Medical Qualification and have experience of Postgraduate Training in Psychiatry. Successful qualification indicates to National Medical Regulators and to potential employers that, on the date of the examination, the exam taker possessed sufficient knowledge of psychiatry according to UEMS criteria to practice as a specialist psychiatrist in Europe. It does not indicate that the holder possesses the necessary skills or behaviors required of a specialist psychiatrist. These attributes must be demonstrated by other means. Further details on the exam and eligibility criteria will be communicated through the channels of EPA, UEMS, and EFPT.

In summary, we successfully leveraged the lessons learned from existing board exams to inform the development and implementation of the first European Board Exam of Psychiatry. This collaborative effort exemplifies a significant advancement in European psychiatric education and sets a precedent for future joint endeavors and medical specialty examinations.
